# Hydrogen bonding in the crystal structure of phurcalite, Ca_2_[(UO_2_)_3_O_2_(PO_4_)_2_]·7H_2_O: single-crystal X-ray study and TORQUE calculations

**DOI:** 10.1107/S2052520620005739

**Published:** 2020-05-27

**Authors:** Jakub Plášil, Boris Kiefer, Seyedat Ghazisaeed, Simon Philippo

**Affiliations:** a Institute of Physics ASCR, v.v.i., Na Slovance 2, Praha 8, 18221, Czech Republic; b Department of Physics, New Mexico State University, Las Cruces, New Mexico NM 88003, USA; c Section Minéralogie, Musée d’Histoire Naturelle, Rue Münster 25, Luxembourg, 2160, Luxembourg

**Keywords:** phurcalite, uranyl phosphate, crystal structure, hydrogen bonding, TORQUE method

## Abstract

The crystal structure of phurcalite, Ca_2_[(UO_2_)_3_O_2_(PO_4_)_2_]·7H_2_O, orthorhombic, *a* = 17.3785 (9) Å, *b* = 15.9864 (8) Å, *c* = 13.5477 (10) Å, *V* = 3763.8 (4) Å^3^, space group *Pbca*, *Z* = 8 has been refined from single-crystal XRD data to *R* = 0.042 for 3182 unique [*I* > 3σ(*I*)] reflections and the hydrogen-bonding scheme has been refined by theoretical calculations based on the TORQUE method. The phurcalite structure is layered, with uranyl phosphate sheets of the phosphuranylite topology which are linked by extensive hydrogen bonds across the interlayer occupied by Ca^2+^ cations and H_2_O groups. In contrast to previous studies the approach here reveals five transformer H_2_O groups (compared to three expected by a previous study) and two non-transformer H_2_O groups. One of the transformer H_2_O groups is, nevertheless, not linked to any metal cation, which is a less frequent type of H_2_O bonding in solid state compounds and minerals. The structural formula of phurcalite has been therefore redefined as {Ca_2_(H_2_^[3]^O)_5_(H_2_^[4]^O)_2_}[(UO_2_)_3_O_2_(PO_4_)_2_], *Z* = 8.

## Abstract 2

The crystal structure of the uranyl-phosphate mineral phurcalite is characterised by extensive hydrogen bonding. It comprises a less common type of H_2_O bonding in solids: a transformer H_2_O unit (with a three-coordinated O atom), which is not directly linked to any metal cation. This study documents the advantage of combining XRD data and TORQUE calculations, which are significantly less demanding of resources than DFT calculations..

## 1. Introduction   

Uranyl phosphates and arsenates represent a group of environmentally important minerals formed during a hydration–oxidation weathering of primary U minerals, mostly uraninite (Finch & Murakami, 1999 ▸; Krivovichev & Plášil, 2013 ▸; Plášil, 2014 ▸). Generally, due to their low solubility products (see *e.g.* Ilton *et al.*, 2010 ▸; Astilleros *et al.*, 2013 ▸; Göb *et al.*, 2013 ▸), they can occur both in the vadose zone of the uranium deposits (Murakami *et al.*, 1997 ▸; Finch & Murakami, 1999 ▸; Plášil *et al.*, 2006 ▸, 2009 ▸; Göb *et al.*, 2013 ▸) and in mine dumps, wastes and tailings (Buck *et al.*, 1996 ▸; Roh *et al.*, 2000 ▸; Fuller *et al.*, 2002 ▸; Catalano *et al.*, 2006 ▸; Cantrell *et al.*, 2011 ▸; Maher *et al.*, 2013 ▸). This makes uranyl phosphate and arsenate minerals essential for controlling U mobility in the environment. Nowadays, more than 50 uranyl phosphates and arsenates are known to occur in nature, some of them being discovered in the past decade (Mills *et al.*, 2008 ▸; Plášil *et al.*, 2010 ▸, 2018 ▸; Pekov *et al.*, 2012 ▸).

The vast majority of uranyl phosphate structures are based on sheets of vertex- and edge-sharing uranyl polyhedra and phosphate tetrahedra. Uranyl phosphate minerals (and arsenates as well) have historically been classified/divided in two major groups, autunite and phosphuranylite groups (Krivovichev & Plášil, 2013 ▸). They essentially differ in details of their topological arrangement of structural units, *i.e.* uranyl-anion topologies. The autunite topology comprises equatorial vertex-sharing between uranyl square bipyramids and phosphate tetrahedra. The phosphuranylite type of structures contains both uranyl pentagonal and hexagonal bipyramids within the sheets that share edges, forming chains that are cross-linked by sharing vertices and edges with phosphate tetrahedra (Burns, 2005 ▸; Lussier *et al.*, 2016 ▸). Mineral phosphuranylite (*s.s.*) contains additionally one extra uranyl square bipyramid located between the sheets making it the 3D framework structure (Demartin *et al.*, 1991 ▸).

Hydrogen bonds are of particular importance for stabilizing the largely hydrated structures of uranyl phosphates and arsenates, thus controlling their thermodynamic stabilities. Consequently, it is important to determine the details of hydrogen bonding in such minerals in order to understand their stability and the mechanisms by which they break down. Nevertheless, the direct determination of the H-atom positions in uranyl-based compounds is challenging, largely due to high absorption of X-rays and small or poorly developed crystals available for the structure analysis. Therefore, the combination of methods, usually comprised of XRD structure determination and density functional theory (DFT) optimization is often adopted (Colmenero *et al.*, 2017 ▸, 2018*a* ▸,*b* ▸,*c* ▸, 2019*a* ▸,*b* ▸,*c* ▸).

Here, we present a complete structure determination, including hydrogen bonding, in a complex structure of uranyl phosphate mineral phurcalite, as determined by combination of X-rays and a recently developed robust, fast real space optimization method (Ghazisaeed *et al.*, 2018 ▸, 2019 ▸).

## 2. Methodology   

### 2.1. Sample   

**Figure 1:**
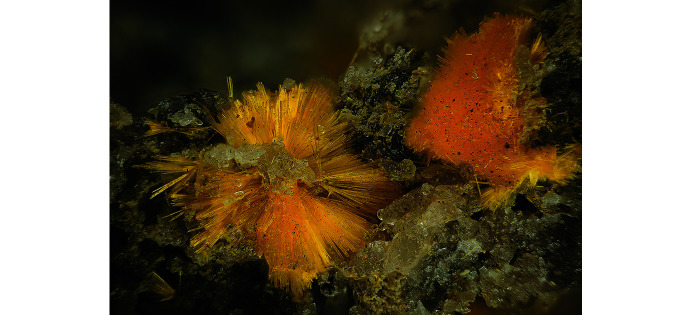
Phurcalite in long-prismatic crystals in quartz-dominant gangue. FOV ∼6 mm across (photo by S. Wolfsried).

The natural specimen used for extraction of phurcalite crystals suitable for X-ray diffraction originates from the Shinkolobwe mine, Shaba province, Democratic Republic of Congo (Africa). Phurcalite forms long-prismatic, needle-like orthorhombic crystals of intense yellow color (Fig. 1 ▸), growing in cavities of quartz with disseminated small crystals of metatorbernite–metazeunerite series of minerals. The specimen has been deposited in the mineral collection of the Musée National d’Histoire Naturelle in Luxembourg (specimen registration number PV025).

### 2.2. Single-crystal X-ray diffraction   

A long-prismatic fragment (0.091 mm × 0.012 mm × 0.009 mm) of phurcalite crystal was selected under a polarized-light microscope and mounted on a glass fiber. The X-ray data collection was done at room temperature with a Rigaku SuperNova single-crystal diffractometer (Mo *K*α radiation from a micro-focus X-ray tube collimated and monochromated by mirror-optics and detected by an Atlas S2 CCD detector). In line with previous structure determinations, phurcalite is found to be orthorhombic, *a* = 17.3785 (9) Å, *b* = 15.9864 (8) Å, *c* = 13.5477 (10) Å, *V* = 3763.8 (4) Å^3^ and *Z* = 8. Integration of the diffraction data, including corrections for background, polarization and Lorentz effects were carried out with the *CrysAlis RED* program (Rigaku, 2019 ▸). An empirical absorption correction was applied to the data in the *Jana2006* software, using spherical harmonics (Petříček *et al.*, 2014 ▸). Crystallographic data and experimental details are given in Table 1 ▸. The structure of phurcalite was solved by the charge-flipping algorithm using the *SHELXT* program (Sheldrick, 2015 ▸). Structure refinement was done using the software *Jana2006* with the full-matrix least-squares refinement based on *F* ^2^. The structure solution revealed positions for all atoms except of hydrogens; those were ascertained from the difference Fourier maps. The H atoms were refined using a mix of soft constraints on O—H distances and with the *U*_eq_ of each H set to 1.2 times that of the donor O atom. The bond-valence sums were calculated following the procedure of Brown (2002 ▸), and using bond-valence parameters taken from Gagné & Hawthorne (2015 ▸).

### 2.3. TORQUE method calculations   

The orientations of the H_2_O molecules were optimized with the TORQUE method, a robust and fast real-space method for determining H_2_O orientations from rotational equilibrium (Ghazisaeed *et al.*, 2018 ▸, 2019 ▸). In all test-cases (haidingerite, Ca[AsO_3_(OH)]·H_2_O, barium chloride monohydrate, BaCl_2_ ˙H_2_O, apophyllite, KCa_4_(Si_4_O_10_)_2_F_1–*x*_(HF)_*x*_·[(H_2_O)_8–*x*_(OH)_*x*_], grimselite, K_3_Na(UO_2_)(CO_3_)_3_·(H_2_O), and kernite, Na_2_B_4_O_6_(OH)_2_·3(H_2_O), the TORQUE-predicted equilibrium H_2_O orientations agreed with available neutron diffraction observations (Ghazisaeed *et al.*, 2018 ▸). In the TORQUE method, the H_2_O molecules are placed such that its oxygen matches the location known from the experiment. In contrast, no prior knowledge of the location of the two hydrogens atoms (per water molecule) is needed. Their locations are obtained from the molecular H_2_O geometry, as described in the TIP3P model [H—O—H angle = 104.52°, and *d*(O—H) = 0.9572 Å; Jorgensen *et al.*, 1983 ▸].

We performed two sets of TORQUE computations to investigate the extent of hydrogen bonding in phurcalite. In the first set, we orient the H_2_O molecules such that they match our X-ray observations as closely as possible. Slight adjustments are needed to account for deviations of *d*(O—H) and H—O—H angle between experiment and water model. More specifically, we place each water molecule in the corresponding experimental H_2_O plane, and adjust the bond geometry, such that the bis­ectors of the H—O—H angle coincide and place the two hydrogen atoms at ±52.26^o^, from the bis­ector at the prescribed molecular O—H distance. With this placement of the H_2_O molecules the complete initial crystal structure of phurcalite is completely specified. Charges for ions in the structural unit are taken from bond-valence analysis (see below), and for H_2_O from the TIP3P model (Jorgensen *et al.*, 1983 ▸). With this information, the torque on the H_2_O molecules is computed and the H_2_O molecules are rigidly rotated about their oxygen ions by a small increment. This torque compution/rigid rotation cycle is continued until the torque is vanishingly small and rotational equilibrium is reached (Ghazisaeed *et al.*, 2018 ▸).

The results address stable and unstable water orientations in the X-ray derived hydrogen bond network. In the second set the H_2_O molecules are oriented randomly while preserving the molecular H_2_O geometry and addresses the (non)uniqueness of the identified rotational equilibria. We optimized 1000 random initial H_2_O orientations and statistically analyzed the similarities and differences of the obtained rotational equilibrium configurations, similar to our previous work (Ghazisaeed *et al.*, 2018 ▸, 2019 ▸, 2020 ▸; Steciuk *et al.*, 2019 ▸). Moreover, we performed an additional TORQUE optimization where the H_2_O initial orientations are chosen as closely as possible to our X-ray refinements.

## 3. Results   

### 3.1. Crystal structure obtained from X-ray diffraction   

**Figure 2:**
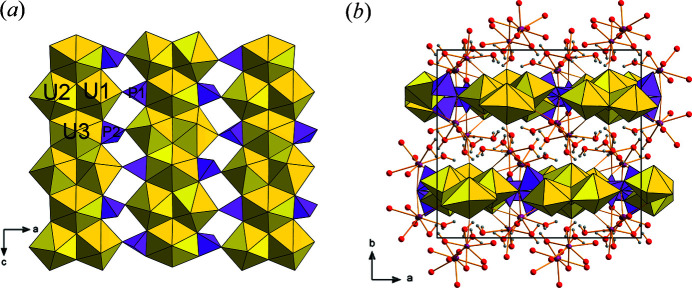
Crystal structure of phurcalite. (*a*) Uranyl phosphate sheet of the phosphuranylite topology containing UO_2_^2+^ coordinated both as UO_7_ (U1 and U2) and UO_8_ bipyramids. (*b*) Stacking of the sheets perpendicular to **b**. Adjacent sheets are linked by an extensive hydrogen bonding network (bonds are omitted for clarity). Color scheme: U is yellow, P is pink, Ca is violet, O is red, H is gray; unit-cell edges are outlined as black solid lines.

The structure of phurcalite as obtained from the current structure determination by X-ray diffraction is in line with previous study done by Atencio *et al.* (1991 ▸). During the current study it was possible to reveal partially some of the positions of the H atoms in the structure and refine them to obtain a reasonable bonding geometry. The structure of phurcalite is based upon uranyl phosphate sheets [Fig. 2 ▸(*a*)] of phosphuranylite topology (Burns, 2005 ▸; Lussier *et al.*, 2016 ▸), with a ring symbol 6^1^5^2^4^2^3^2^ (Krivovichev & Burns, 2007 ▸); with hexagons of the topology occupied by U^6+^. Unlike sheets of other members of the phosphuranylite group (Piret & Declercq, 1983 ▸; Piret *et al.*, 1988 ▸; Demartin *et al.*, 1991 ▸; Dal Bo *et al.*, 2017 ▸), the sheet in phurcalite does not contain H atoms either as OH or as molecular H_2_O. The composition of the sheets are hydrogen free, [(UO_2_)_3_O_2_(PO_4_)_2_]^4–^, and stacked perpendicular to the [010] direction in phurcalite [Fig. 2 ▸(*b*)]. Between adjacent sheets two independent Ca sites are located. The Ca1 is linked to seven ligands, including four O of the H_2_O groups, two uranyl O atoms (of the U3 and U2) and one O atom of the P2 tetrahedron. The Ca2 site is surrounded by eight ligands including five O atoms from H_2_O groups, two uranyl O atoms (one to the U1 and one to the U2 polyhedra) and one bond to to P2 tetrahedron. Two of the H_2_O (with Wyckoff 8*c* = two H_2_O pfu) are shared between Ca1 and Ca2 (O13 and O20) that form dimers of the composition {Ca_2_(H_2_O)_7_O_6_}. The detailed analysis of the hydrogen bonding is given below.

### 3.2. Hydrogen bonding as revealed from both X-rays and TORQUE   

The stereochemical details of the hydrogen bonding as revealed from X-rays and TORQUE calculations are given in Table 2 ▸. There are seven independent O atoms corresponding to H_2_O groups in the structure of phurcalite: following the XRD structure determination, H_2_O is expected to belong to sites O16, O17, O19, O20, O21, O22, O23. However, the detailed orientation of O17 could not be resolved due to insufficiently resolved difference Fourier maxima from the X-ray data.

### 3.3. Discussion – hydrogen bonding   

X-ray structure refinements and results from TORQUE provide strong evidence for extensive hydrogen bonding in phurcalite. In contrast to the results of our X-ray diffraction refinements, TORQUE successfully identified reasonable H_2_O hydrogen bond arrays for all seven water sites, including O17 (Table 2 ▸).

**Figure 3:**
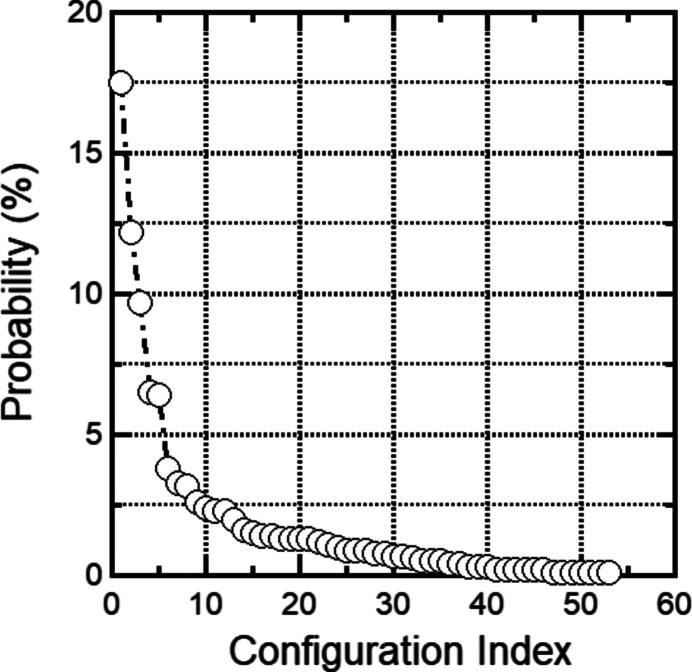
Probabilities for the 53 non-equivalent TORQUE identified H_2_O equilibrium orientations in phurcalite.

Bond-valence analysis shows that calculated sums of bond-valence at the sites are within a few percent of expected oxidation states of all elements in phurcalite (Table 3 ▸). Therefore, we chose the corresponding formal charges for all non-H_2_O toms for the TORQUE simulations. We obtained rotational equilibria for 1000 randomly initialized configurations. In order to compare more directly to X-ray data, we identified structures as equivalent, if the closest acceptor for all seven H_2_O sites for two configurations is the same. We TORQUE-optimized 1000 randomly chosen initial H_2_O orientations and found 53 geometrically distinct O—H⋯A environments (H_2_O rotational equilibrium orientations), with occurrences that range from 0.1% to 17.5% (see Fig. 3 ▸). However, only six of the seven-site H_2_O environments are predicted to have an occurrence probability of 6% or higher (with a joint probability of 52.3%, Fig. 3 ▸). This observation suggests that a comparatively small number of O—H⋯*A* environments likely capture a significant fraction of the stereochemical variability, at least in phurcalite. The stereochemical results for the average seven-site model for the highest probability O—H⋯*A* environment (17.5%) are shown in Table 2 ▸, the TORQUE predicted hydrogen acceptor sites are listed in Table 4 ▸, and the corresponding hydrogen positions are listed in Table 5 ▸. The reported standard deviations were obtained from the analysis of the TORQUE-predicted equilibrium orientations that belong to an equivalent set. For example, for the highest probability configuration, 175 equilibrium orientations were averaged, and the corresponding standard deviations were computed. If we analyze the probability of orientations for each site, we find that all seven water orientations appear either with the highest or second highest probability (Table 4 ▸). This observation that not every site belongs to the highest probability orientations demonstrates that local and global rotational equilibrium do not necessarily coincide, and correlated changes in the water array must be taken into account during data analysis. A comparison of the stereochemistry of the water positions determined by X-ray diffraction and the 53 equilibrium H_2_O orientations shows no simultaneous match for all seven sites. Complete O20 and O22 stereochemistry matches occur in our library with probabilities of 4.2% and 38.5%, respectively (Table 4 ▸). Partial matches exist for O17, O19, O21 and O23, and no match is found for O16. This result suggests that the X-ray derived water stereochemistry does not correspond to a rotational equilibrium state. In order to explore whether this conclusion is due to sampling, we initialized TORQUE close to our X-ray-derived H_2_O positions (while preserving the predefined H_2_O geometry of the TIP3P water model, see method section for details on hydrogen placement); we find again significant re-bonding of hydrogen, partial matches can be found for O19, O21, O22 and O23, while complete re-bonding is predicted for O16, O17 and O20 (Table 4 ▸, optimized hydrogen positions are listed in Table 6 ▸. However, in contrast to the X-ray derived H_2_O array we find a simultaneous match for all seven water sites among the 53 equilibrium orientations with a probability of 3.8%, ranked #6 among the 53 distinct rotational equilibrium orientations (Fig. 3 ▸). Therefore, it is unlikely that the X-ray hydrogen positions correspond to an accidentally unsampled rotational equilibrium state, and uncertainties can be more likely attributed to simultaneous rotations of several H_2_O molecules.

The X-ray O16 water site has no match among the TORQUE determined 53 equilibrium H_2_O orientations, while all other sites at least show a partial match. For O16, the X-ray observations suggest (Table 2 ▸) hydrogen bonding to O19 (water) and O20 (water). In contrast, TORQUE predicts bonding to O10 (U3) and O23 (water). The driving force for re-bonding is H32 which is only 1.87 Å from Ca2 in the refined X-ray data, closer than any of its oxygen ligands. The corresponding Ca—H electrostatic repulsion provides a driving torque for water re-orientation, and in rotational equilibrium we find *d*(H32–Ca2) = 3.02 Å, an increase of ∼60%. Therefore, the X-ray O16 stereochemistry is predicted to be unstable, and we note that the TORQUE-optimized O16 water orientation appears in our library with a probability of 8.8% (Table 4 ▸).

**Figure 4:**
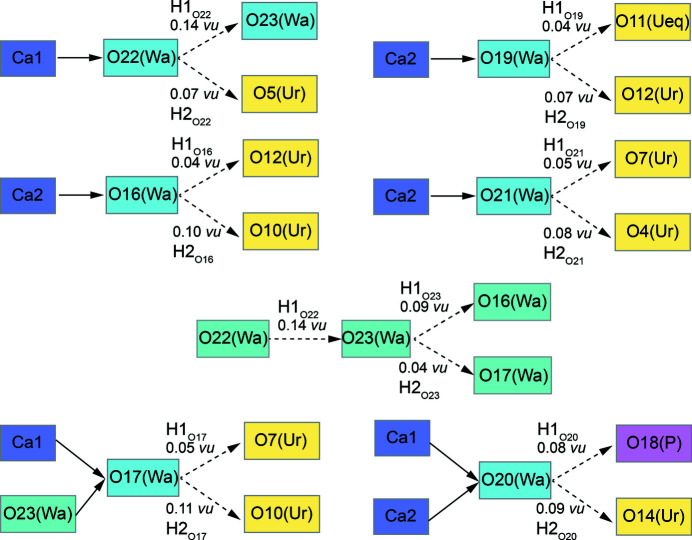
Bonding scheme concerning interstitial H_2_O groups in phurcalite. (Ur) – uranyl apical O atom, (*U*_eq_) – uranyl equatorial O atom, (Wa) – H_2_O molecule, (P) – O atom of the PO_4_ group, bond-strengths given in valence-units (vu).

X-ray diffraction was unable to identify reasonable hydrogen bonding for O17. The origin of this inability may be explained by TORQUE-predicted re-bonding, the X-ray observations suggest hydrogen bonding (Table 2 ▸) with O19 (water) and O23 (water). However, we find hydrogen atoms only ∼1.5 Å from O17H1 and O17H2, distances comparable to the intramolecular H–H distance. Therefore, H–H repulsion induces water rotation and a new stereochemistry to O7(U2) and O10(U3), which we find for the TORQUE-optimized X-ray orientations, as well as for the highest probability model in our library and corresponds to the highest probability O17 orientation (66.3%, Tables 2 ▸ and 4 ▸). Therefore, TORQUE successfully describes a rotational equilibrium state for O17, that could not be resolved from our X-ray diffraction results. The discussion of possible hydrogen-bonding arrangements in phurcalite has been used in the theoretical bond-valence studies (Schindler & Hawthorne, 2008 ▸) focused on interactions between anionic (*i.e.* Lewis bases) and cationic (*i.e.* Lewis acids) parts of the structures of hydrated oxysalts. Their conclusions, which they found on the basis of the bond-valence theory (Brown, 2002 ▸, 2009 ▸; Hawthorne, 2012 ▸, 2015 ▸), were that phurcalite contains three transformer H_2_O groups (having a corresponding O atom as three-coordinated; for details check Fig. 5 in Schindler & Hawthorne, 2008 ▸), three non-transformer H_2_O groups (having a corresponding O atom as four-coordinated) and one non-transformer H_2_O group not bonded to any cations; the composition of the interstitial complex was expressed as {Ca_2_(H_2_^[3]^O)_3_(H_2_^[4]^O)3(H_2_O)_1_} (Schindler & Hawthorne, 2008 ▸). Our study advances the understanding of H_2_O complexes and their interactions with the surrounding crystal framework in phurcalite. From the scheme given in Fig. 4 ▸ it is possible to simply read off that there are five transformer H_2_O groups (with corresponding O atom being three-coordinated); one bonded to Ca1 atom (O22) three others bonded to Ca2 atom (O16, O19, O21) and an additional one, O23, which is not linked to the metal cation (see below). Furthermore, there are two non-transformer H_2_O groups (with corresponding O atom being four-coordinated). First one, O17, is linked to Ca1 site, nevertheless accepts also one weak hydrogen bond from H1_O19_. Second one, O20, is shared between Ca1 and Ca2 atoms. Finally, the O23 atom belongs to the transformer H_2_O group, with no linkage to any metal cation; O23 receives one hydrogen bond from H1_O22_ and transform it into two hydrogen bonds, *via* H1_O23_ and H2_O23_, therefore the O23 is three-coordinated. The magnitude of strength of two corresponding hydrogen-bonds (H1_O23_ + H2_O23_ = 0.13 vu) match the initial strength of the hydrogen-bond accepted by O23 (0.14 vu). To summarize, the interstitial complex in phurcalite can be expressed as {Ca_2_(H_2_^[3]^O)_5_(H_2_^[4]^O)_2_}. Therefore, the structural formula of phurcalite is {Ca_2_(H_2_^[3]^O)_5_(H_2_^[4]^O)_2_}[(UO_2_)_3_O_2_(PO_4_)_2_], *Z* = 8.

## 4. Conclusions   

The structure of the mineral phurcalite (calcium uranyl phosphate heptahydrate) is stabilized by an extensive network of hydrogen bonds. Phurcalite is unique among uranyl phosphates in that it shows a high Ca:U ratio (2:3) (for instance mineral autunite has 1:2) and its structure displays an unusual hydrogen bonding scheme. Structure data obtained from a XRD experiment and theoretical calculations (TORQUE) indicate that the structure of phurcalite contains a rare functional type of H_2_O group in the interlayer which is not linked to any metal cation directly, as it accepts one hydrogen bond from an adjacent H_2_O group. This H_2_O group thus splits the incident bond-strength (represented by one incoming hydrogen bond) into two weaker hydrogen bonds. Therefore it is a transformer H_2_O group with a three-coordinated O atom. Our study advances our understanding of hydrogen bonding in complex uranyl minerals and shows the synergy of experiment and theory provides new insights into the complex hydrogen bonding in uranyl phosphates and the role of H_2_O groups in complex oxysalt minerals. In summary, it is likely that the rare hydrogen bonding topology in phurcalite is responsible for its low abundance in nature.

## Supplementary Material

Crystal structure: contains datablock(s) global, I. DOI: 10.1107/S2052520620005739/yh5003sup1.cif

Structure factors: contains datablock(s) I. DOI: 10.1107/S2052520620005739/yh5003Isup2.hkl

CIF TORQUE. DOI: 10.1107/S2052520620005739/yh5003sup3.txt

CIF TORQUE 2. DOI: 10.1107/S2052520620005739/yh5003sup4.txt

CCDC reference: 1999464

## Figures and Tables

**Table 1:** Experimental details

Crystal data	
Chemical formula	Ca_2_(H_2_O)_6_[(UO_2_)_3_O_2_(PO_4_)_2_]·(H_2_O)
*M*_r_	1238.3
Crystal system, space group	Orthorhombic, *Pbca*
Temperature (K)	293
*a*, *b*, *c* (Å)	17.3785 (9), 15.9864 (8), 13.5477 (10)
*V* (Å^3^)	3763.8 (4)
*Z*	8
No. of reflections for cell measurement	3639
Radiation type, wavelength (Å)	Mo *K*α, 0.71073
θ range (°) for cell measurement	4.0–29
μ (mm^−1^)	26.58
Crystal size (mm)	0.09 × 0.01 × 0.01

Data collection	
Diffractometer	SuperNova, Dual, Cu at zero, AtlasS2
Absorption correction	Empirical (using intensity measurements) (*JANA2006*)
*T*_min_, *T*_max_	0.982, 1
No. of measured, independent and observed [*I* > 3σ(*I*)] reflections	20 436, 4721, 3182
*R*_int_	0.067
(sin θ/λ)_max_ (Å^−1^)	0.698

Refinement on *F* ^2^ by *Jana2006*	
*R* (obs), *R* (all)	0.042, 0.0647
*wR* (obs), *wR* (all)	0.080, 0.074
*S* (all)	1.22
No. of reflections	4721
No. of parameters	198
No. of restraints	21
H-atom treatment	All H-atom parameters refined
Δρ_max_, Δρ_min_ (e Å^−3^)	3.58, −3.41

Computer programs: *CrysAlis PRO* 1.171.38.43 (Rigaku, 2015 ▸), *Jana2006* (Petříček *et al.*, 2014 ▸).

**Table 2:** Hydrogen-bond geometry as obtained from XRD data and TORQUE calculations Left: XRD; Right: TORQUE. For TORQUE, we list we list the highest probability joint seven-site model (17.5%). For details, see text.

XRD					Torque				
*D*—H⋯*A*	*D*—H (Å)	H⋯*A* (Å)	*D*⋯*A* (Å)	*D*—H⋯*A* (°)	*D*—H⋯*A*	*D*—H (Å)	H⋯*A* (Å)	*D*⋯*A* (Å)	*D*—H⋯*A* (°)
O16—H1_O16_⋯O19	0.95 (8)	1.98 (9)	2.762 (14)	139 (7)	O16—H1_O16_⋯O12	0.957	2.286	3.126	146.1
O16—H2_O16_⋯O20^iii^	0.94 (9)	2.39 (8)	3.248 (14)	151 (7)	O16—H1_O16_⋯O10	0.955	1.884	2.801	160.0
O17—H1_O17_⋯O23^xii^	0.95 (5)	1.89 (7)	2.763 (13)	153 (7)	O17—H1_O17_⋯O7	0.957	2.160	2.821	125.1
O17—H2_O17_⋯O19^x^	0.94 (7)	2.36 (9)	2.944 (13)	120 (9)	O17—H2_O17_⋯O10	0.956	1.846	2.782	165.3
O19—H1_O19_⋯O17^x^	0.95 (8)	2.02 (9)	2.944 (13)	162 (8)	O19—H2_O19_⋯O12	0.959	2.251	3.179	162.4
O19—H2_O19_⋯O11^v^	0.95 (7)	1.92 (8)	2.809 (10)	155 (8)	O19—H1_O19_⋯O11	0.957	2.043	2.809	135.7
O20—H1_O20_⋯O8^xi^	0.94 (8)	2.34 (8)	3.123 (11)	141 (7)	O20—H1_O20_⋯O18	0.959	2.009	2.966	175.3
O20—H2_O20_⋯O22^xi^	0.94 (6)	2.18 (4)	3.074 (14)	159 (8)	O20—H2_O20_⋯O14	0.956	1.969	2.870	156.4
O21—H1_O21_⋯O12^xv^	0.95 (9)	2.37 (10)	3.241 (12)	153 (7)	O21—H2_O21_⋯O7	0.959	2.185	3.090	156.8
O21—H2_O21_⋯O5^ii^	0.95 (9)	2.41 (11)	2.892 (12)	111 (8)	O21—H2_O21_⋯O4	0.959	1.981	2.921	165.8
O22—H1_O22_⋯O23	0.94 (5)	1.90 (8)	2.700 (13)	141 (9)	O22—H1_O22_⋯O23	0.952	1.769	2.698	164.5
O22—H2_O22_⋯O5^vii^	0.95 (8)	2.11 (8)	3.033 (12)	162 (8)	O22—H1_O22_⋯O5	0.961	2.076	3.033	174.4
O23—H1_O23_⋯O17^xiv^	0.95 (6)	2.13 (9)	2.763 (13)	123 (8)	O23—H1_O23_⋯O16	0.957	1.806	2.751	168.9
O23—H2_O23_⋯O7	0.94 (9)	2.38 (9)	3.268 (12)	157 (8)	O23—H2_O23_⋯O17	0.959	1.911	2.764	146.7

Symmetry codes: (ii) *x* − ½, −*y* + ½, −*z* + 1; (iii) *x* − ½, *y*, −*z* + ½; (v) *x*, −*y* + ½, *z* + ½; (vi) *x* + ½, *y*, −*z* + ½; (vii) −*x* + 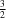, *y* + ½, *z*; (x) –*x* + 1, −*y* + 1, −*z* + 1; (xi) −*x* + 2, −*y* + 1, −*z* + 1; (xii) −*x* + 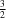, −*y* + 1, *z* − ½; (xiv) –*x* + 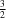, −*y* + 1, *z* + ½; (xv) −*x* + 1, *y* + ½, −*z* + ½.

**Table 4:** Summary of all site occurrences among the 1000 configurations Nearest oxygen acceptor sites for the two hydrogens are shown in parenthesis. Bold and underlined are TORQUE-predicted sites that agree with our X-ray diffraction experiment. Detailed hydrogen positions for the random TORQUE seven-site model (probability 17.5%), and EXP + TORQUE model are given in Table 5 ▸ and Table 6 ▸, respectively.

*N* = 1000	EXP	EXP + TORQUE seven-site model	Random TORQUE seven-site model (*P* = 17.5%)	Probability of occurrence					
O16	(19+20)	(10+23)	(10+12)	91.2% (10+12)	8.8% (10+23)				
O17	(19+23)	(7+10)	(7+10)	66.4% (7+10)	33.6% (10+**23**)				
O19	(11+17)	(**11**+12)	(**11**+12)	90.3% (**11**+12)	9.7% (12+**17**)				
O20	(8+22)	(14+18)	(14+18)	82.2 (14+18)	11.2% (4+18)	4.2% (**8**+**22**)	2.4% (4+13)		
O21	(5+12)	(**5**+7)	(4+7)	49.3% (**5**+7)	46.7% (4+7)	4.0% (4+**5**)			
O22	(5+23)	(**5**+14)	(**5**+**23**)	55.2% (**5**+14)	38.5% (**5** +**23**)	4.1% (4+**5**)	2.0% (14+**23**)	0.2% (**5**+20)	
O23	(7+17)	(**17**+22)	(16+**17**)	39.0% (16+22)	33.8% (16+**17**)	26.8% (**17**+22)	0.2% (16+16)	0.1% (11+16)	0.1% (16+21)

**Table:** 

	U1	U2	U3	Ca1	Ca2	P1	P2	ΣBV								
O1	0.61	0.65	0.66					1.93								
O2		0.53	0.26			1.19		1.98								
O3	0.61	0.58	0.70					1.89								
O4			1.65	0.24				1.88								
O5		1.65			0.16			1.81								
O6		0.55	0.33			1.23		2.11								
O7		1.66		0.18				1.84								
O8	0.44		0.21				1.21	1.87								
O9		0.44			0.28		1.19	1.92								
O10			1.71					1.71								
O11	0.53					1.33		1.85								
O12	1.78							1.78								
O13				0.31	0.33	1.30		1.93								
O14	1.69				0.13			1.81								
O15	0.37		0.42				1.19	1.97								
O16					0.28			0.28								
O17				0.34				0.34								
O18				0.42			1.39	1.80								
O19					0.29			0.29								
O20				0.25	0.20			0.45								
O21					0.31			0.31								
O22				0.34				0.34								
O23								0.00								
ΣBV	6.02	6.07	5.94	2.07	1.97	5.05	4.98									

**Table:** 

+H-bonds	H1_O16_	H2_O16_	H1_O17_	H2_O17_	H1_O19_	H2_O19_	H1_O20_	H2_O20_	H1_O21_	H2_O21_	H1_O22_	H2_O22_	H1_O23_	H2_O23_	ΣBV
O1															1.93
O2															1.98
O3															1.89
O4										0.08					+1.88 = 1.96
O5												0.07			+1.81 = 1.88
O6															2.11
O7			0.05						0.05						+1.84 = 1.94
O8															1.87
O9															1.92
O10		0.10		0.11											+1.71 = 1.92
O11					0.04										+1.85 = 1.89
O12	0.04					0.07									+1.78 = 1.89
O13															1.93
O14								0.09							+1.81 = 1.90
O15															1.97
O16	0.91	0.92											0.12		+0.28 = 2.23
O17			0.91	0.91										0.10	+0.34 = 2.26
O18							0.08								+1.80 = 1.88
O19					0.91	0.91									+0.29 = 2.11
O20							0.91	0.91							+0.45 = 2.27
O21									0.91	0.91					+0.31 = 2.13
O22											0.91	0.90			+0.34 = 2.15
O23											0.14		0.91	0.91	1.96
ΣBV	0.95	1.02	0.97	1.03	0.95	0.98	0.99	1.00	0.96	0.99	1.06	0.97	1.04	1.01	

The bond-valence parameters taken from Gagné & Hawthorne (2015 ▸).

**Table 6:** TORQUE-predicted fractional positions for hydrogen, if the method is initialized close to the corresponding refined X-ray positions

TORQUE	Site	*x*	*y*	*z*
O16	H1_O16_	0.54435	0.48978	0.37795
	H2_O16_	0.57984	0.40381	0.36667
O17	H1_O17_	0.70755	0.46820	0.50272
	H2_O17_	0.69837	0.42671	0.40297
O19	H1_O19_	0.40669	0.48376	0.45977
	H2_O19_	0.41338	0.40528	0.52172
O20	H1_O20_	0.99650	0.52464	0.37201
	H2_O20_	0.99494	0.43179	0.35014
O21	H1_O21_	0.31019	0.55300	0.31527
	H2_O21_	0.28558	0.46597	0.28448
O22	H1_O22_	0.90000	0.57427	0.64744
	H2_O22_	0.81632	0.57138	0.61656
O23	H1_O23_	0.84478	0.48224	0.71565
	H2_O23_	0.82143	0.48288	0.82330

**Table 5:** TORQUE-predicted average fractional positions and standard deviations for the TORQUE-optimized X-ray hydrogen bond scheme for the highest probability seven-site model (17.5%) A standard deviation of (0) signifies that is smaller than the last displayed digit.

TORQUE	Site	*x*	*y*	*z*
O16	H1_O16_	0.53179 (2)	0.44109 (2)	0.43288 (1)
	H2_O16_	0.57752 (1)	0.40415 (1)	0.35006 (3)
O17	H1_O17_	0.71263 (1)	0.46332 (2)	0.50366 (0)
	H2_O17_	0.69911 (0)	0.42675 (0)	0.40202 (1)
O19	H1_O19_	0.39835 (1)	0.48544 (0)	0.45829 (1)
	H2_O19_	0.40868 (2)	0.41112 (0)	0.52627 (1)
O20	H1_O20_	0.99421 (2)	0.52502 (1)	0.37683 (2)
	H2_O20_	0.99646 (2)	0.43307 (1)	0.35018 (2)
O21	H1_O21_	0.28743 (1)	0.52979 (2)	0.31847 (1)
	H2_O21_	0.32157 (0)	0.53923 (0)	0.21624 (0)
O22	H1_O22_	0.86960 (19)	0.5092 (3)	0.66145 (4)
	H2_O22_	0.82376 (6)	0.58223 (11)	0.62206 (1)
O23	H1_O23_	0.90269 (2)	0.49392 (1)	0.80610 (5)
	H2_O23_	0.81637 (2)	0.48353 (6)	0.81419 (11)
